# Senescence as a dictator of patient outcomes and therapeutic efficacies in human gastric cancer

**DOI:** 10.1038/s41420-021-00769-6

**Published:** 2022-01-10

**Authors:** Lulin Zhou, Zubiao Niu, Yuqi Wang, You Zheng, Yichao Zhu, Chenxi Wang, Xiaoyan Gao, Lihua Gao, Wen Zhang, Kaitai Zhang, Gerry Melino, Hongyan Huang, Xiaoning Wang, Qiang Sun

**Affiliations:** 1 School of Medicine, Nankai University, 94 Weijin Road, Tianjin, 300071 China; 2 Institute of Biotechnology, Research Unit of Cell Death Mechanism, Chinese Academy of Medical Sciences, 20 Dongda Street, Beijing, 100071 China; 3 Department of Oncology, Beijing Shijitan Hospital of Capital Medical University, 10 TIEYI Road, Beijing, 100038 China; 4 Department of Immunology, National Cancer Center/National Clinical Research Center for Cancer/Cancer Hospital, Chinese Academy of Medical Sciences and Peking Union Medical College, Beijing, 100021 China; 5 State Key Laboratory of Molecular Oncology, Department of Etiology and Carcinogenesis, National Cancer Center/National Clinical Research Center for Cancer/Cancer Hospital, Chinese Academy of Medical Sciences and Peking Union Medical College, Beijing, 100021 China; 6 Department of Experimental Medicine, TOR, University of Rome “Tor Vergata”, Rome, 00133 Italy; 7 DZNE German Center for Neurodegenerative Diseases, 53127 Bonn, Germany; 8 Institute of Geriatrics, The second Medical Center, Beijing Key Laboratory of Aging and Geriatrics, National Clinical Research Center for Geriatric Diseases, Chinese PLA General Hospital, Beijing, 100853 China; 9 School of Laboratory Medicine and Biotechnology, Southern Medical University, Guangzhou, 510515 China

**Keywords:** Gastric cancer, Chemotherapy, Cancer immunotherapy, Prognostic markers, Senescence

## Abstract

Senescence is believed to be a pivotal player in the onset and progression of tumors as well as cancer therapy. However, the guiding roles of senescence in clinical outcomes and therapy selection for patients with cancer remain obscure, largely due to the absence of a feasible senescence signature. Here, by integrative analysis of single cell and bulk transcriptome data from multiple datasets of gastric cancer patients, we uncovered senescence as a veiled tumor feature characterized by senescence gene signature enriched, unexpectedly, in the noncancerous cells, and further identified two distinct senescence-associated subtypes based on the unsupervised clustering. Patients with the senescence subtype had higher tumor mutation loads and better prognosis as compared with the aggressive subtype. By the machine learning, we constructed a scoring system termed as senescore based on six signature genes: ADH1B, IL1A, SERPINE1, SPARC, EZH2, and TNFAIP2. Higher senescore demonstrated robustly predictive capability for longer overall and recurrence-free survival in 2290 gastric cancer samples, which was independently validated by the multiplex staining analysis of gastric cancer samples on the tissue microarray. Remarkably, the senescore signature served as a reliable predictor of chemotherapeutic and immunotherapeutic efficacies, with high-senescore patients benefited from immunotherapy, while low-senescore patients were responsive to chemotherapy. Collectively, we report senescence as a heretofore unrecognized hallmark of gastric cancer that impacts patient outcomes and therapeutic efficacy.

## Introduction

Gastric cancer (GC) is the third leading cause of cancer death and the fifth most common cancer globally [1]. The chemotherapy is one of the major therapeutic strategies for GC patients. However, there is considerable heterogeneity in the treatment response and clinical outcomes among cancer patients despite in similar clinical or pathologic conditions [2], which underscores the need for novel predictive markers other than clinical stages and pathohistological classifications. With the rapid advance in the high-throughput sequencing technologies, molecular subtypes of GC were established by The Cancer Genome Atlas (TCGA) including microsatellite instable (MSI), genomically stable (GS), chromosomal instability (CIN), Epstein-Barr virus (EBV) associated, and hypermutated-single-nucleotide variants (HM-SNV) [3], as well as by the Asian Cancer Research Group (ACRG) comprising microsatellite stable (MSS)/epithelial–mesenchymal transition (EMT), MSI, MSS/p53+, and MSS/p53− [4]. However, these defined classifications face huge obstacles in the clinical translation and limitations in relevant cancer patients.

Cancer immunotherapy such as immune checkpoint inhibitors (ICIs) elicits durable antitumor responses in multiple solid cancers. However, only a fraction of patients with cancer respond to immunotherapy, calling for biomarkers for better clinical management [5]. Although it was revealed that a wide spectrum of tumor features, such as genomic instability described as MSI or tumor mutational burden (TMB) status and tumor-infiltrating immune cells in tumor microenvironment (TME), were associated with immunotherapy sensitivity in preclinical and clinical settings [5–7], it is still a daunting challenge to predict the treatment responses of chemotherapy and immunotherapy simultaneously, and to make the appropriate therapeutic options.

Cellular senescence, defined as the state of cell-cycle arrest, can be triggered by severe interior or exterior insults such as oncogenic activation or chemotherapeutic DNA damage [8, 9]. Cellular senescence was believed to be a failsafe program for the organism by excluding deleterious cells from further expansion, and thereby suppresses tumorigenesis in a cell-autonomous setting [10–12]. On the other hand, senescent cells can secrete a plethora of cytokines and growth factors including interleukin-6 (IL-6) and IL-8, termed as senescence-associated secretory phenotype (SASP), whereby establish an immunosuppressive, inflammatory and catabolic microenvironment to promote tumor growth and resistance to chemotherapy [12–15]. Conversely, the SASP factors can also trigger senescence in an autocrine or paracrine fashion to impose an antitumor effect [16, 17]. Therefore, it is imperative to elaborate the impacts of the senescence on tumors for better cancer prevention and therapy. Furthermore, there exist a wide spectrum of attributes that distinguish senescent cells from their normal counterparts, such as prominent changes in the cell size and morphology, increased expression of the CDK inhibitors CDKN2A, and enhanced DNA damage response (DDR), but the unique and specific markers for senescent cells are still absent [8, 18]. In fact, though preclinical studies involving the cell experiments and animal models have revealed the extensive effects of senescence on cancer, our knowledge about the senescent characteristic in tumor samples of patients is still very rudimentary. In particularly, whether the senescent feature in patients with cancer could serve as a biomarker to guide clinical prognosis and treatment remain unknown.

**Fig. 1:**
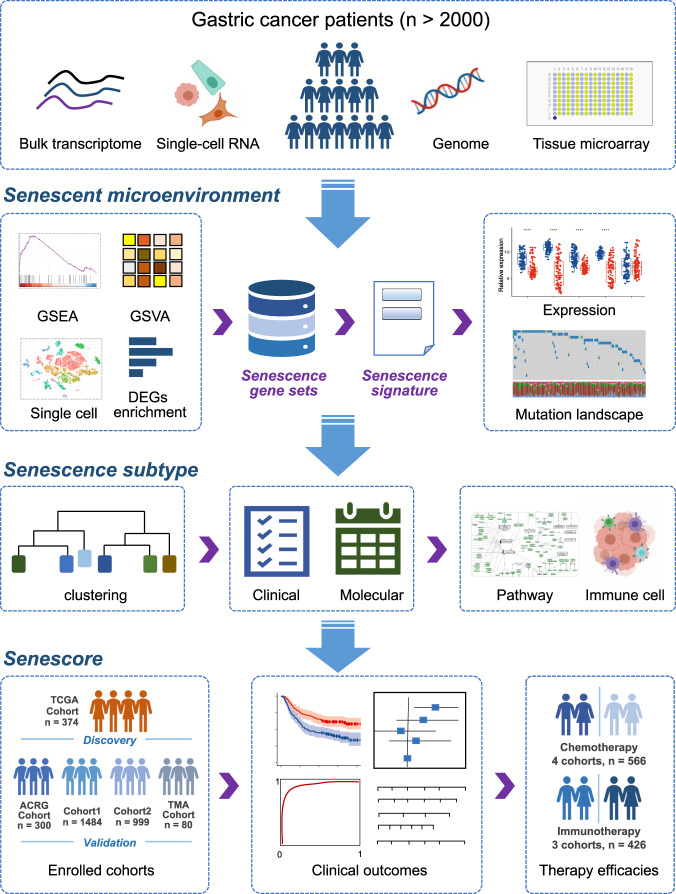
Overview of the work flow in this study. Multiomics data ranging from bulk and single-cell transcriptome, genome to protein expression derived from more than 2000 gastric cancer patients were included in this pipeline analysis. Multiple bioinformatics algorithms including GSEA, GSVA, and differential expression analysis were applied to dissect senescent microenvironment and identify the senescence signature in gastric cancer. Moreover, the senescence subtype was established through the unsupervised clustering, and further was parsed in clinical and biological relevance. Finally, the senescence scoring system, senescore, was developed and validated for clinical prognosis and therapy efficacies in wide spectrum of patient cohorts.

In this study, we performed the comprehensive analysis of senescent features within TME in multiple GC cohorts, and further identified the senescence subtypes and dissected its association with clinical and molecular features, as well as the immune cell infiltration and signaling pathway activation. Finally, we demonstrated that senescore, a senescence scoring system of tumor, exhibited robust predictive powers not only for the patients outcomes but also for the efficacies of different therapeutic strategies including adjuvant chemotherapy and immunotherapy (Fig. 1).

## Results

### Identification of senescence as a hallmark of GC

**Fig. 2:**
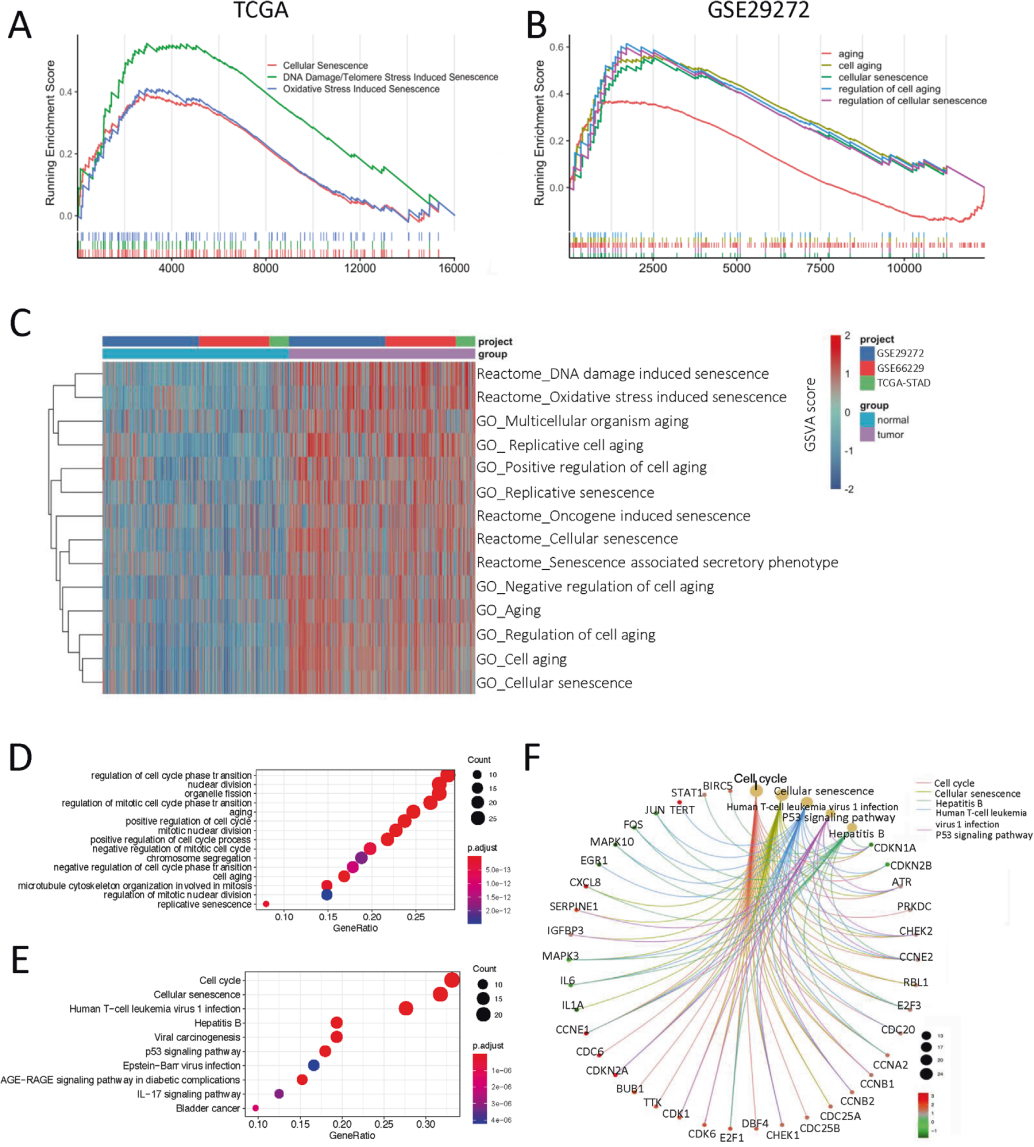
Identification of the senescence character in gastric cancer. **A** GSEA plot showed that senescence-associated pathways were significantly enriched in GC relative to normal tissues in TCGA-STAD cohort. Multiple pathway enrichment results were indicated by corresponding colors. **B** GSEA plot exhibited the enriched GO terms associated with senescence based on GC versus normal gastric tissues in GSE29272 cohort. The different GO terms were displayed by corresponding colors. **C** GSVA enrichment analysis showing the quite different senescence features between GC and their paired adjacent normal tissues in multiple datasets. Heatmap displaying the GSVA score of senescence pathways and GO terms. Dot plots showing the significant enrichment results for senescence-associated genes in GO term (**D**, top 15) and pathway (**E**, top 10). The dot size and color represented the gene count and the weighted Fisher’s *p* value, respectively. **F** The interaction network exhibiting the top five enriched pathways and their contained genes. The dot size of the pathways represented the gene count. The color of genes indicated the fold change of gene expression between GC and normal tissues in TCGA-STAD cohort.

To elucidate the senescence character of GC, we comprehensively explored the transcriptomic data in multiple datasets of GC. In TCGA-STAD cohort, the results of gene set enrichment analysis (GSEA) showed that those biological pathways indicative of cellular senescence, including cellular senescence (NES = 1.63, *p* < 0.01), DNA damage/telomere stress induced senescence (NES = 1.98, *p* < 0.01), and oxidative stress induced senescence (NES = 1.58, *p* < 0.01), were significantly enriched in the GC over normal gastric tissues (Fig. 2A). And Gene Ontology (GO) about cellular senescence was also significantly enriched in GC compared with normal tissues (Fig. S1A). Similarly, consistent results for senescence were observed in the another independent cohort (Fig. 2B, Fig. S1B). Moreover, the gene set variation analysis (GSVA) between GC and their paired adjacent normal tissues in three datasets showed GC qualified with markedly activation in multifarious senescence pathways and GO terms (Fig. 2C). These results suggested senescence as a prominent hallmark of GC.

To investigate the genes impacting senescence in GC, we firstly screened the differently expressed genes (DEGs) between GC and no-tumor adjacent tissues in TCGA-STAD dataset. It was shown that 3225 DEGs were identified, including 1455 upregulated and 1770 downregulated genes (Fig. S1C, D), and the upregulated genes were markedly enriched in the cellular senescence pathway (Fig. S1E). Then, we acquired senescence genes based on all the gene sets associated with senescence in Molecular Signatures Database (MSigDB) [19] (Table S1). Combined the DEGs with senescence genes, 103 senescence-associated genes in GC were identified. GO and pathway enrichment analysis of these genes was conducted, showing significantly enriched biological processes comprising aging, cell cycle and cellular senescence (Fig. 2D, E). As shown in Fig. 2F, the interaction network between top five enriched pathways and genes encompassed the specific marker for cellular senescence (CDKN2A) and immune-associated genes (e.g., CXCL8 and IL-6).

### Single-cell profiling of senescence within TME in GC

**Fig. 3:**
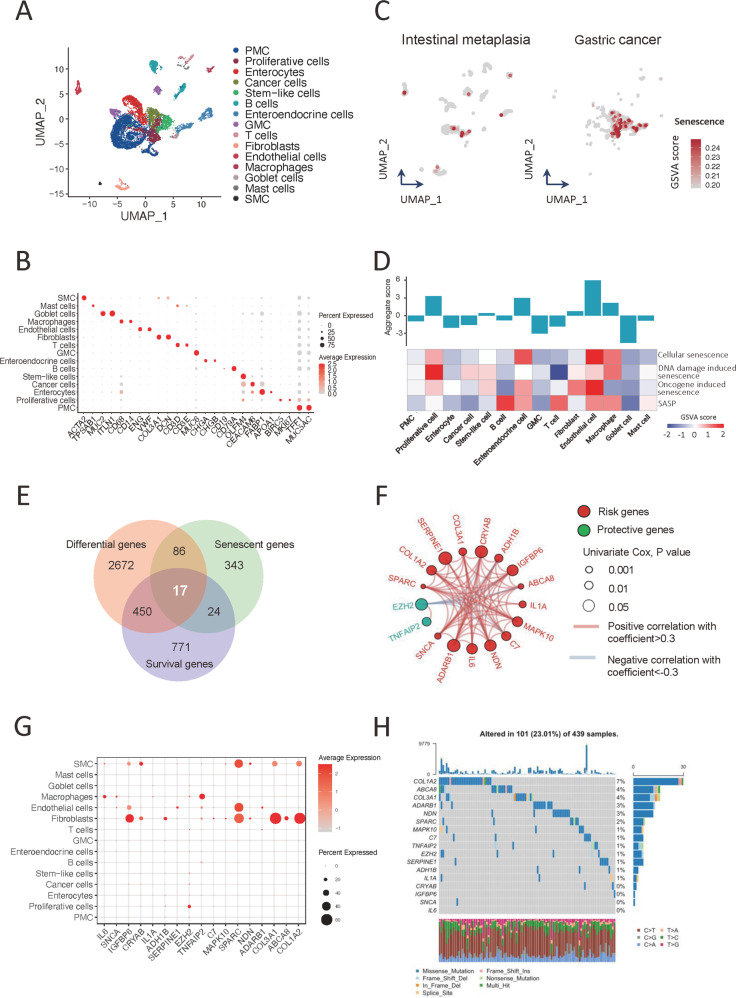
Single-cell profiling of the senescent microenvironment in gastric cancer. **A** UMAP plot showed single-cell transcriptomic profiling of the cell types from gastric normal tissues and GC. **B** Dot plot represented the expression of canonical marker genes of each cell type in gastric normal tissues and GC. **C** Cell senescence signature enrichment displayed in different cells from normal and tumor gastric tissues. **D** Cell senescence signatures analyses of GSVA scores for different cell types in GC. **E** Venn diagram showed that the senescence core genes were established by overlapping among the DEGs, senescence genes and survival genes. **F** The interaction among CSGs in GC. The risk genes and protective genes were marked with red and green, respectively. The circle size represented *p* value of univariate Cox regression analysis. The lines connecting genes showed their interactions based on correlation. Positive correlation was marked with red and negative correlation with gray. **G** Dot plot showed the expression of CSGs of each cell type in GC. **H** The mutation landscape of CSGs in patients from TCGA-STAD cohort. Each column represented individual patients. The upper barplot showed TMB, and the number on the right presented the mutation frequency in each gene. The barplot in right showed the proportion of each variant type. The stacked barplot below showed fraction of conversions in each sample. PMC pit mucous cells, GMC gland mucous cells, SMC smooth muscle cells, DEGs differently expressed genes.

To further dissect the senescence characteristics within GC tissues at single-cell level, we investigated the single-cell transcriptome of 19,042 cells from normal and cancerous gastric tissues (Fig. 3A, B, Fig. S2A). In agreement with the results from the bulk analysis above, more cells with significant activation of cellular senescence pathway were identified in GC as compared with those in normal gastric tissues (Fig. 3C, Fig. S2B). Interestingly, in GC, certain cell types consisting of endothelial cells, enteroendocrine cells, proliferative cells, and macrophages as well as fibroblasts, but not the cancer cells, are highly enriched in various senescence pathways (Fig. 3D), suggesting these cell types, rather than the cancer cells, may primarily contribute to the senescence features in GC.

To identify the key genes associated with senescence in GC, we integrated significantly DEGs, the survival-associated genes and senescence genes derived from senescence gene sets, and finally established the core senescence genes (CSGs) of GC (Fig. 3E, F). At single-cell level, CSGs were expressed with higher proportions in cells of TME such as endothelial cells, fibroblasts, and macrophages (Fig. 3G), which is in line with above pathway analysis.

Furthermore, we assessed the incidence of copy number variations (CNV) and somatic mutations of 17 CSGs in GC using genomic data. It was indicated that *COL1A2* gene exhibited the highest mutation frequency followed by *ABCA8*, while the investigation of CNV alteration frequency showed that a prevalent CNV amplification occurred in *COL1A2*, *C7*, *ABCA8*, as well as *SERPINE1* gene (Fig. 3H, Fig. S2C, D). Although the gene-expression changes were associated with the genomic transformation, especially the alteration of CNV (Fig. S2C, E), the complexity of genomics and heterogeneity of tumor led to the incomplete consistency in the landscape of genomic and expressional alterations, highlighting that expression characteristics of CSGs played a considerably pivotal role in GC.

### The identification of senescence subtypes based on the CSGs

**Fig. 4:**
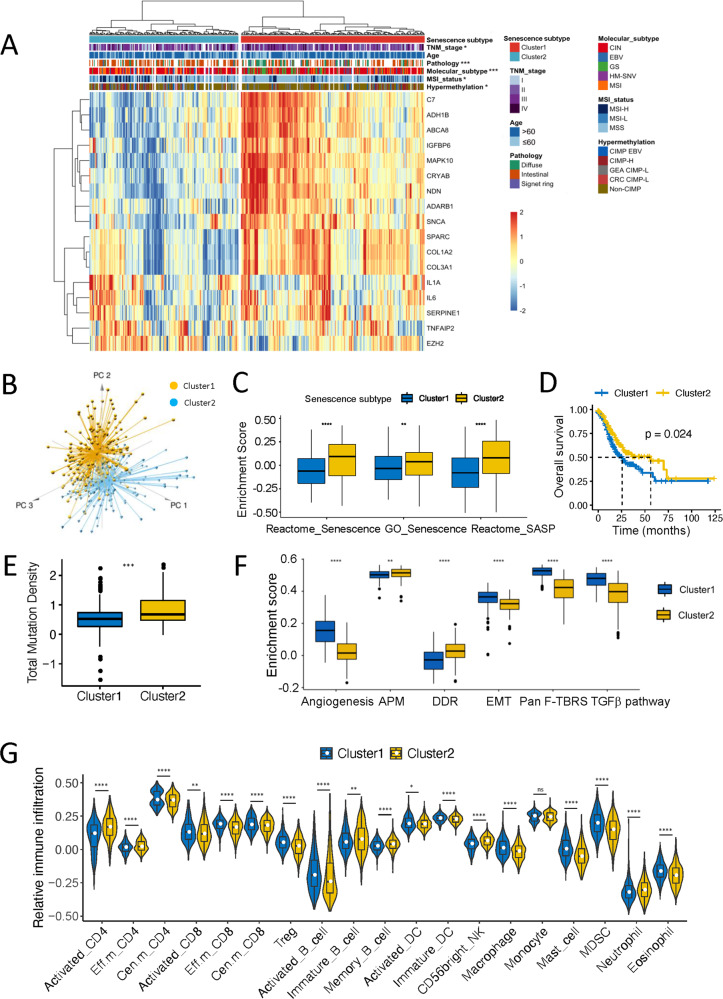
Development of senescence subtypes based on senescence core genes. **A** Unsupervised clustering of patients derived from the TCGA-STAD cohort based on CSGs to classify patients into two senescence subtypes. TNM stage, age pathological classification, molecular subtype, MSI status, and hypermethylation category were shown above the heatmap. **B** Principal component analysis for the expression profiles to distinguish two clusters in TCGA-STAD cohort. Cluster 1 was marked with yellow and cluster 2 was marked with blue. **C** GSVA analysis for senescence gene sets indicating the significant distinction of senescence features in two subtypes. **D** Survival analyses for the two subtypes in TCGA-STAD cohort. Kaplan–Meier curves showed a significant survival difference between two clusters. *p* value was calculated by log-rank test. **E** The significant distinction in total mutation density between senescence subtypes. **F** Differences in pathway activation including angiogenesis, APM, DDR, EMT, Pan F-TBRS, and TGFβ pathway between two subtypes. In **C**, **E**, and **F**, the box represents the interquartile range, the horizontal line in the box is the median, and the whiskers represent 1.5 times the interquartile range. **G** Violin plot depicting the abundance of each cell type in different clusters in the validation cohort 1. In **C** and **E**–**G**, the asterisks represented the statistical *p* value (Wilcoxon’s rank-sum test, *p < 0.05; **p < 0.01; ***p < 0.001). MSI microsatellite instability, HM-SNV hypermutated-single-nucleotide variants, CIMP CpG island methylator phenotype, MSS microsatellite stable, SASP senescence-associated secretory phenotype, APM antigen processing machinery, DDR DNA damage repair response, EMT epithelial–mesenchymal transition, Pan F-TBRS pan-fibroblast TGFβ response signature, Eff.m effector memory, Cen.m central memory, Treg regulatory T cells, DC dendritic cells, NK natural killer, MDSC myeloid-derived suppressor cells.

In order to decipher the underlying biological characteristics of the senescence phenotype, the unsupervised hierarchical clustering analysis was performed firstly in the discovery cohort of TCGA-STAD based on the expression of CSGs. Two senescence-associated clusters were identified (Fig. 4A), and principal component analysis (PCA) indicated the significant distinction existed on the transcriptional profile between the two subtypes (Fig. 4B, Fig. S3A). Except for *EZH2*, *TNFAIP2*, and *IL1A*, the remaining genes exhibited higher expression in cluster 1 than those in cluster 2 (Fig. S3B). Furthermore, functional enrichment analysis showed that cluster 2 displays significantly higher activation in senescence pathways, suggesting cluster 2 was associated with senescence subtype (Fig. 4C). Survival analysis showed that cluster 2 was associated with longer overall survival (OS) (Fig. 4D), which was independently validated in a pooled cohort of 1484 patients (Fig. S3C, D). Interestingly, the higher mutation density was observed in cluster 2 in comparison with cluster 1 in TCGA dataset (Fig. 4E).

Moreover, the senescence subtypes were parsed in the clinical and molecular features in TCGA cohort. The results showed that majority of patients with early clinical stage (stage I) were in cluster 2 (Fig. S4A). And no significant distribution difference was found between groups stratified by the age of patients (Fig. S4B), which was probably resulted from the little age gap among patients (Table S2). We also found that tumors in cluster 1 presented poorer differentiation and were enriched in the diffuse and signet-ring histological classification (Fig. S4C), suggesting cluster 1 was associated with the more aggressive subtype. With regard to the molecular subtypes of TCGA project [3], the GS subtype was mainly concentrated in the cluster 1, while the subtypes of MSI and EBV-positive substantially appeared in the cluster 2 (Fig. S4D). In consistent with the molecular subtype, cluster 2 was higher in the proportion of MSI tumors by the MSI status analysis and the clonal deletion score for quantificational CIN than those in cluster 1 (Fig. S4E, F).

In addition, we analyzed the canonical biological processes associated with TME, indicating that the cluster 2 displayed activation of DDR, antigen processing and presentation (APM), which may result from the higher mutation load (Fig. 4F) [20]. Furthermore, we then explored the characteristics of the immune cell infiltration in 1484 GC patients in the validation cohort 1. The results showed that cluster 1 showed significant increases in the infiltration of antitumor immune cells such as the activated CD8^+^ T cells and central memory CD4^+^ T cells, as well as protumor immune cells such as the regulatory T cells, and myeloid-derived suppressor cells. By contrast, cluster 2 appeared high infiltration of activated CD4^+^ T cells, CD56 bright natural killer cells (NK), and memory B cells (Fig. 4G). These results indicated that the senescence subtypes were associated with distinct features of TME.

### Construction of a senescence scoring model

**Fig. 5:**
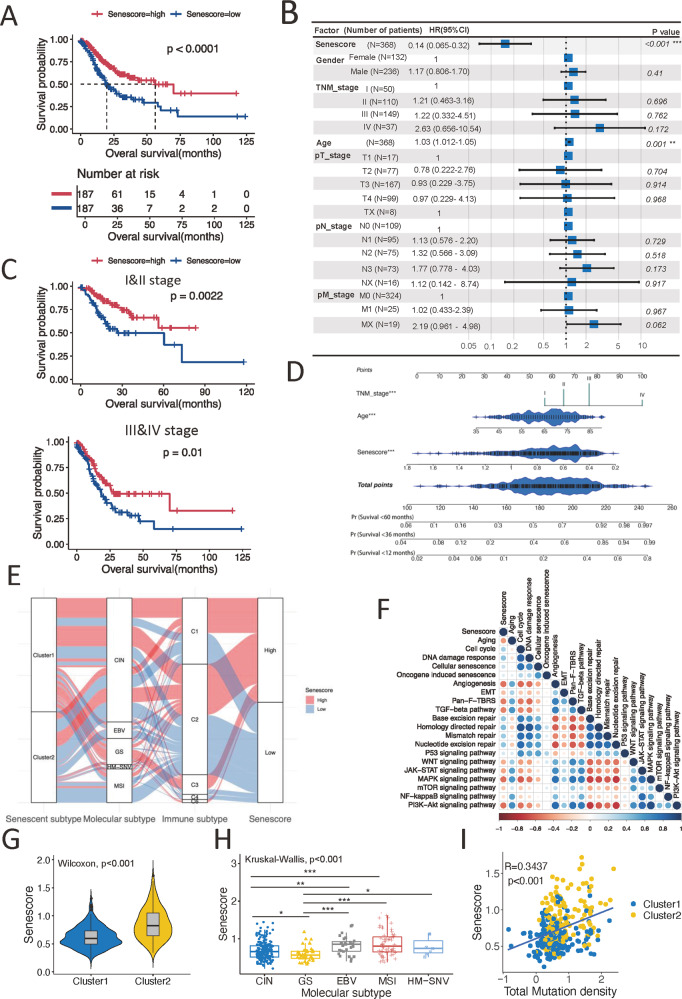
Construction of the senescence score with clinical utility. **A** Kaplan–Meier curves indicated the significant survival difference between high- and low-senescore groups in TCGA-STAD cohort. Log-rank test, *p* < 0.0001. **B** Multivariate Cox regression analysis for senescore and other clinical characteristics with OS in TCGA-STAD cohort shown by the forest plot. **C** Kaplan–Meier curves showing patients with stage I/II (upper panel) or stage III/IV (lower panel) was further grouped into different survival by senescore. Log-rank test, *p* value was indicated. **D** Nomogram was developed in the TCGA-STAD cohort based on the senescore, age, and TNM stage to predict 1-year, 3-year, and 5-year outcomes. **E** Alluvial diagram showing the mutuality of the senescence subtypes, molecular subtypes, immune subtypes, and senescore in TCGA-STAD cohort. **F** Correlations between senescore and myriad biological processes in TCGA-STAD cohort using Spearman analysis. Negative correlation was marked with red and positive correlation with blue. The size of dots represents the value of correlation coefficient. **G** Violin plot displaying the differences of senescore between senescence subtypes in TCGA-STAD cohort. The Wilcoxon rank-sum test was used to compare the statistical significance between two groups. **H** The box plot showing the distinctions of the senescore in different molecular subtypes. The asterisks represented the statistical *p* value (the Wilcoxon rank-sum test, *p < 0.05; **p < 0.01; ***p < 0.001). The Kruskal–Wallis test was applied to evaluate significant differences among all five variables. **I** Scatterplot showing the correlations of the senescore with mutation density. R coefficient represents of Pearson’s correlation. *p* value was from the Pearson correlation test. The shaded area represents 95% confident interval. The blue and yellow colors of the dots respectively represent the cluster 1 and cluster 2.

The senescence phenotype acted nonnegligible roles in shaping the TME and markedly affected the clinical prognosis. However, these analyses were only based on the patient population and could not accurately predict the senescence character in individual patients. Therefore, we sought to construct a scoring system to quantify the senescence level in tumor tissues for individual patient with GC. In the TCGA-STAD cohort, we constructed a senescence scoring model, termed “senescore,” by using six senescence signature genes (ADH1B, IL1A, SERPINE1, SPARC, EZH2, and TNFAIP2) identified with the least absolute shrinkage and selection operator (LASSO) Cox regression algorithm (Fig. S5A, B). The survival analysis demonstrated patients with high senescore were correlated with better outcomes in TCGA-STAD cohort (log-rank test, *p* < 0.0001; median OS 19.6 months versus 56.2 months, Fig. 5A and Table S3). Furthermore, when the senescore was evaluated as a continuous variable with the multivariate Cox regression model, the senescore was determined to be an independent and robust prognostic factor (HR, 0.14; 95% CI, 0.065–0.32; *p* < 0.001; Fig. 5B). More importantly, when patients were stratified with TNM clinical stages, the senescore successfully separated patients with distinct clinical prognosis at the same disease stage (Fig. 5C). These findings suggested that the senescore reserved its prognostic relevance even after classic clinicopathologic prognostic features have been taken into accounts. Therefore, for better clinical utility, we integrated senescore with the age and clinical stages, two independent prognostic factors identified above, to generate a comprehensive nomogram (Fig. 5D). The prognostic capacity of the nomogram was also demonstrated by the area under the curve (AUC) of the time-dependent receiver operating characteristic (ROC) curve with a value of 0.794 at 5 years (Fig. S5C). Furthermore, the calibration curves of the model for the possibility of OS at 3 years and 5 years closely approximated the observed estimates, manifesting the accurate predictive ability (Fig. S5D, E).

To better illustrate the feature of senescore, the overview of the connection among the senescence subtypes, molecular subtypes, immune subtypes, and the senescore of individual patients in TCGA cohort was depicted by the alluvial diagram (Fig. 5E). And the correlations between the senescore and varying biological processes suggested that senescore was positively associated with the activation of DDR, senescence, and DNA damage repair pathway (Fig. 5F). The senescore was significantly higher in cluster 2 subtype than that in cluster 1 (Fig. 5G). Whereas for the established molecular subtypes, the GS subtype had the lowest median senescore, while the EBV subtype and the MSI subtype showed significantly correlated with higher senescore (Fig. 5H). Similarly, the senescore was also associated with the MSI status, corresponding MSI-high (MSI-H) to the highest senescore (Fig. S5F). Furthermore, the senescore was positively correlated with the mutation density (Fig. 5I). In addition, senescore signature was associated with histological classification, showing patients with intestinal GC had highest senescore (Fig. S5G). These findings suggested that the senescore was closely associated with clinical characteristics of GC patients and may serve as a prognostic biomarker.

### Validation of senescore to effectively predict patient outcomes

**Fig. 6:**
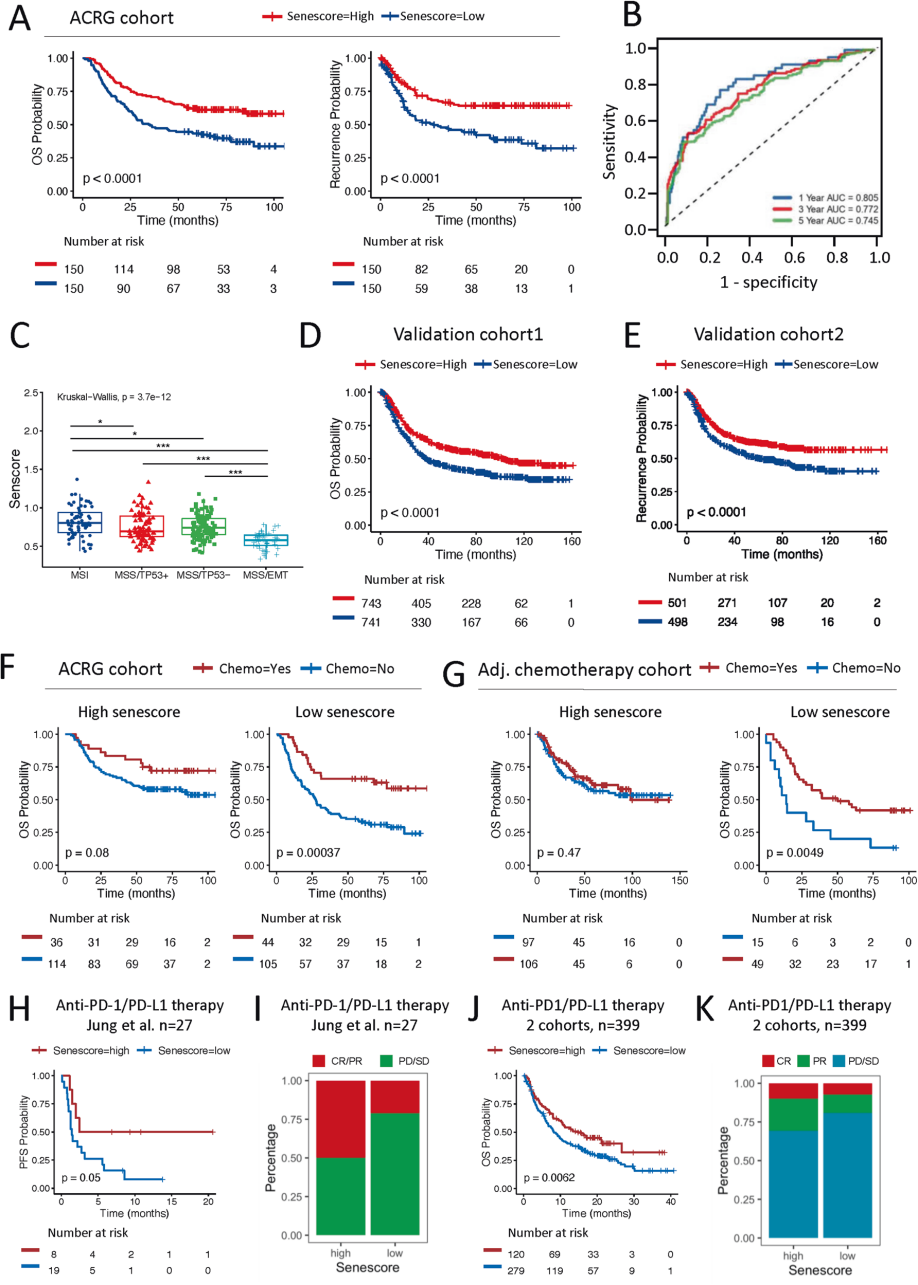
Validation of the senescore for clinical prognosis and therapeutic efficacy. **A** Kaplan–Meier plots showing the differences in OS (left) and RFS (right) between the high- and low-senescore groups in the ACRG cohort. *p* value was calculated by log-rank test. **B** Time‐dependent ROC curve for the validation of nomogram in the ACRG cohort. **C** Differences of the senescore among molecular subtypes in the ACRG cohort (the Wilcoxon rank-sum test, *p < 0.05; **p < 0.01; ***p < 0.001). The Kruskal–Wallis test was applied to evaluate significant differences among four variables. **D** Kaplan–Meier plot indicating the distinctions in patients’ OS between high- and low-senescore groups in the validation cohort 1. **E** Kaplan–Meier plot displaying the predictive value of the senescore for patients’ RFS in the validation cohort 2. **F** Kaplan−Meier plots of GC patients of the high- and low-senescore group in ACRG cohort. The data in each group were analyzed according to whether patients received adjuvant chemotherapy or not. **G** Survival analyses for patients in adjuvant chemotherapy cohort were stratified by both the senescore and treatment with adjuvant chemotherapy using Kaplan–Meier curves. **H** Survival analyses for low and high-senescore patients in an independent anti-PD-L1 therapy cohort using Kaplan–Meier curves. **I** The proportion of patients with response to PD1/PD-L1 blockade immunotherapy in the patients with high or low senescore. **J** Kaplan–Meier curves showed survival differences of OS between low and high-senescore patients in the integrated cohort of anti-PD1/PD-L1 therapy. **K** The proportion of patients with the therapy response to PD1/PD-L1 blockade immunotherapy in the high or low-senescore groups. In **D**–**H** and **J**, *p* value was calculated by log-rank test. OS overall survival, RFS recurrence-free survival, Chemo chemotherapy, PFS progress-free survival, CR complete response, PR partial response, PD progressive disease, SD stable disease.

To confirm the robustness of the senescore in different populations, we further explored ACRG cohort that was provided with the comprehensive clinical information. Similar with the TCGA cohort, the survival analysis revealed that the high senescore was associated with the longer OS as well as the recurrence-free survival (RFS) (*p* < 0.001; hazard ratio, 0.12 [95% CI, 0.04–0.33]) (Fig. 6A). And the prognostic nomogram demonstrated an impressive predicting ability for OS (1-year AUC = 0.81, 3-year AUC = 0.77, 5-year AUC = 0.75) as indicated by the time-dependent ROC curve analysis (Fig. 6B). Moreover, the significant differences of the senescore were also observed among molecular subtypes in ACRG cohort. Rather, the MSI subtype showed the highest senescore, while the MSS/EMT subtype had the lowest senscore (Fig. 6C). In addition, the histological classification also displayed the obvious differences of the senescore values (Fig. S6A), which was in consistent with the results in TCGA-STAD cohort. Additional validation of the senescore model was further confirmed in the validation cohort 1 (*n* = 1484) for OS and in the validation cohort 2 (*n* = 999) for RFS, as well as all GC patients in our study (Fig. 6D, E, Fig. S6B). Next, the prognostic power of senescore was examined in a wide spectrum of gastrointestinal tumors, and significantly validated in colorectal cancer and esophageal cancer, respectively, suggesting senescore signature may be conserved in other types of cancer (Fig. S6C–G).

### Senescore predicts the efficacies of chemotherapy and immunotherapy

Considering that cellular senescence could be induced by DNA damage drug, we explored whether senescore, the senescence scoring model, could predict the treatment efficiency of chemotherapy. In ACRG cohorts with available adjuvant chemotherapy information, patients were divided into high- and low-senescore group and the difference in OS and RFS was independently assessed. Adjuvant chemotherapy was found to markedly improve the OS rate and RFS rate in patients with low senescore (*p* = 0.00037), while patients with the high senescore showed only a moderate benefit from adjuvant chemotherapy (*p* = 0.08, Figs. 6F, Fig. S7A). Furthermore, patients with GC in another three cohorts of adjuvant chemotherapy were categorized into high- and low-senescore group. And significant survival difference was observed in the low-senescore group, but not in the high-senescore group (Figs. 6G, Fig. S7B), which was consistent with the results in ACRG cohort. This result suggests that the patients with low-senescore were more inclined to gain benefits from the chemotherapy.

However, in contrast to chemotherapy, patients with high senescore, especially the highest quartile senescore, exhibited significantly prolonged OS in an independent anti-PD-L1 therapy cohort (*n* = 348) (Fig. S7C, D) [21]. In addition, patients responsive to anti-PD-L1 therapy displayed higher senescore (Fig. S7E) and senescore in this immunotherapy cohort was positively related with tumor mutation load (Fig. S7F), suggesting patients with high senescore was associated with better immunotherapy efficiency. Similarly, patients with high senescore in a small anti-PD1/PD-L1 cohort (*n* = 27) [22] displayed longer progress-free survival (PFS) (Fig. 6H), as well as higher overall response (50% versus 21%) compared with low senescore group (Fig. 6I). Similar findings were independently confirmed in a cohort of large size (*n* = 399), in which the patients of urothelial tumors [21] and melanoma [23] receiving anti-PD1/PD-L1 therapy were combined, where the high-senescore group displayed a significantly longer OS (Fig. 6J), and a higher proportion of overall response (31% versus 19%) (Fig. 6K). These results indicated that senescore might serve as a potential biomarker helping the clinicians to select appropriate patients for either chemotherapy or immunotherapy.

### Validation of senescore at translational level

**Fig. 7:**
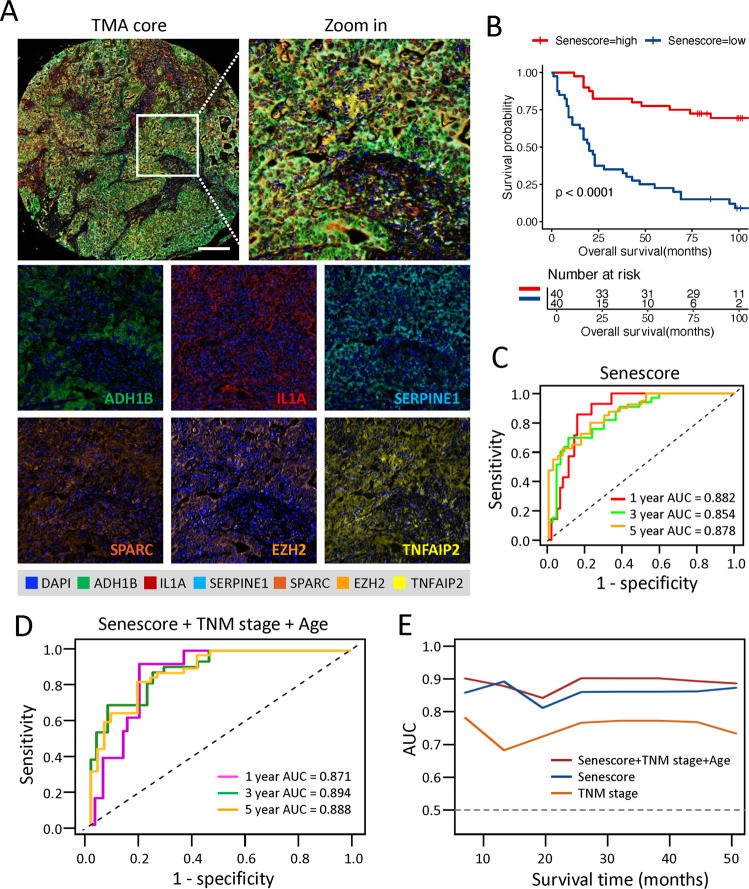
Multiplex immunofluorescence immunohistochemistry profiling of the senescore in tissue microarray of GC. **A** Representative multiplex immunofluorescence immunohistochemistry images showed the protein expression patterns of ADH1B (green), IL1A (red), SERPINE1(cyan), SPARC (orange), EZH2 (pink), and TNFAIP2 (yellow) in GC tissues. DAPI: blue; scale bar: 200 μm. **B** Kaplan–Meier plot showed the significant difference of OS between high- and low-senescore groups in the tissue microarrays of GC. Log-rank test, *p* value was indicated. **C** ROC curve showed the predictive capability of senescore for overall survival of GC patients. **D** ROC curves of the senescore combined with clinical stage and age for predicting 1-year, 3-year, and 5-year survival. **E** Comparison of AUC of the time-dependent ROC curve among nomogram containing senescore, clinical stage and age, and only senescore or clinical stages.

To further validate our model at translational level for the purpose of clinical pathology, a sophisticated method of multiplex immunofluorescence histochemistry, which allows simultaneous detection of multiple target proteins, was employed to analyze the protein expression of the six senescence signature genes identified above on a GC tissue microarray (TMA, Fig. 7A). Among these senescence signatures, four molecules, each by their own, were associated with clinical prognosis (Fig. S8A–F). Consistent with transcriptional analysis above, higher senescore in TMA cohort was significantly associated with longer OS (HR = 0.28, 95% CI: 0.17–0.45; *p* < 0.0001) (Fig. 7B). Furthermore, the ROC curve was conducted to evaluate the sensitivity and specificity of the senescore, and it illustrated that the AUC values was 0.882, 0.854, and 0.878 for 1, 3, and 5 years, respectively (Fig. 7C). In addition, ROC curve analysis also demonstrated the forceful predictive ability of the prognostic nomogram integrating senescore with clinical stage and age (Fig. 7D). More importantly, the senescore displayed stronger predictive value for OS in comparison with TNM stages, while the nomogram model combining the clinical stage and age with senescore only slightly elevated the predictive accuracy of the patients’ prognosis as compared with senescore alone (Fig. 7E). Collectively, senescore is promising in predicting clinical prognosis by feat of the immunofluorescence histochemistry strategy.

## Discussion

In this study, we identified senescence as a hallmark of GC that is associated with distinctive TME and genomic alterations. Therefore, the identification of the senescence subtypes provides an insightful perspective on tumors from another angle. For example, The senescence subtype (cluster 2) showed the features of MSI as well as CIN. It is explained that tumors with genomic instability have more somatic mutations, which can induce cellular senescence by the pathway of DDR [24]. Finally, it was revealed that cluster 2 presented significant survival advantage, highlighting the activation of cellular senescence pathway may play a beneficial role in the clinical prognosis of patients with GC, and likely other types of cancers as well [25].

Furthermore, we established the senescence scoring algorithm to evaluate the senescence level of tumor tissues, termed as senescore. Our results demonstrated that the senescore is of importance in clinical relevance. Integrated analysis revealed that the senescore is a robust prognostic biomarker in a vast number of cancer patients. More importantly, the senescore is able to serve as an independent prognostic factor. Furthermore, the senescore is associated with different established molecular subtypes, histological subtypes, and genomic alterations. For example, high senescore is related with the MSI-H status, EBV infection, and higher TMB. Several studies have demonstrated that patients with MSI-H and EBV-positive tumors as well as high TMB are more inclined to dramatic responses to immunotherapy such as ICIs [26–28]. Consistent with our analysis above, it was indicated that high senescore is more likely to gain benefit from the immunotherapy. Moreover, the technology of multiplex immunofluorescence histochemistry demonstrated a good validation of senescore in robustly predicting patient outcomes, which is promising in bridging the senescence scoring system to the clinical practice in near future.

More importantly, subset analysis of patients with available chemotherapy data strongly suggested that GC patients with low senescore were more cline to gain benefit from the chemotherapy. Previous studies have demonstrated that cellular senescence could increase the drug resistance and side effects of the chemotherapy [29–31], suggesting high senescore associated with the activation of the senescence program that might blunt the desirable effects of the chemotherapy.

In conclusion, this is the first comprehensive senescent analysis of GC, leading to the identification of senescence subtypes that are associated with significantly different TME and survival outcomes. Our analysis demonstrated that the senescore can be used to identify not only patients at different risk levels for the OS as well as RFS but also patients who would benefit from adjuvant chemotherapy or immunotherapy. Impressively, the association between the senescore and immunotherapy effects, though being relatively weak due to the limit of available patient samples, was significant, which encourages further evaluation in a larger size of patients taking immunotherapy. Nevertheless, the validation of our findings in a wide spectrum of patient cohorts, and the findings that the senescence features reflect biological and clinical characteristics associated with sensitivity or resistance to the therapy, would pave a way for developing more rational therapy recommendations and promoting personalized cancer therapy.

## Materials and methods

### Data collection and processing

We retrospectively acquired the sample information of 2290 patients of GC from 12 eligible GC datasets from TCGA and Gene Expression Omnibus (GEO) database. In addition, transcriptomic and clinical data of 1324 patients with other digestive system tumors including colorectal cancer, esophageal cancer, liver cancer, and pancreatic cancer were obtained from TCGA database. And an independent dataset of 579 patients with colon cancer was downloaded from GEO database. Overall, the information of all the datasets enrolled in this study was summarized in Table S4.

For TCGA dataset, the raw count expression data and the normalized RNA sequencing data (FPKM value) were downloaded from TCGA Data Portal using the TCGAbiolinks R package [32]. Then, FPKM values of the normalized RNA sequencing data were transformed into transcripts per kilobase million (TPM) values for analysis. Moreover, the microarray gene-expression data from GEO database were obtained and processed through two strategies. For microarray data of Affymetrix platform, we downloaded the raw “CEL” files and adopted the robust multiarray average algorithm for background adjustment and quantile normalization, and the median polish algorithm for final summarization of oligonucleotides per transcript in the affy R package. For microarray data from other platforms, the processed normalized matrix data were directly downloaded by the GEOquery R package [33]. Batch effects from technological biases were corrected using the ComBat algorithm of sva package when necessary [34]. All the R packages in this study were performed in the R software (version 3.6.1, https://www.r-project.org).

### Patient population and clinical information

In this study, we retrospectively collected 2290 patients of GC from the curated public databases for survival analysis. The TCGA-STAD cohort as the exploratory cohort included 374 patients with detailed clinical information (Tables S2 and S3). The validation cohorts embodied an independent ACRG cohort (GSE66229) and two large pooled cohorts (validation cohort 1 and validation cohort 2). The validation cohort 1 (*n* = 1484) used as OS analysis was integrated with identifiers GSE26899, GSE26901, GSE28541, GSE13861, GSE66229, GSE15459, GSE34942, GSE84437, GSE57303, and GSE29272. And the validation cohort 2 (*n* = 999) as RFS analysis was comprised of identifiers GSE26253, GSE26899, GSE26901, GSE13861, and GSE66229. OS was established as the time from surgery to death or to the last follow-up time, and RFS was defined as the date from surgery to the first verified recurrence. Clinical data of patients in these cohorts was summarized in Table S2.

Furthermore, adjuvant chemotherapy data were available for the patients in the ACRG cohort including 80 patients receiving postoperative adjuvant chemotherapy [4]. With regard to another validation cohort of the adjuvant chemotherapy, 267 patients who underwent gastrectomy as primary treatment at three cohorts (KUGH cohort under GSE26899, KUCM cohort under GSE26901 and YUSH cohort under GSE13861 datasets) were enrolled, including 155 patients who had received standard adjuvant chemotherapy (either single-agent 5-fluorouracil or a combination of 5-fluorouracil and cisplatin/oxaliplatin, doxorubicin, or paclitaxel) (Table S5) [35]. We collected three independent datasets of patients treated with immunotherapies with available transcriptomics data (Table S6). For the Mariathasan et al.’s anti-PD-L1 therapy cohort, the data of 348 patients with metastatic urothelial tumors treated with the anti-PD-L1 agent were obtained from IMvigor210CoreBiologies R package (http://research-pub.gene.com/IMvigor210CoreBiologies/) [21]. The raw count data were normalized by implementing trimmed mean of M values method and transformed with the “voom” function in the limma package [36]. For another anti-PD1/PD-L1 therapy cohort of lung cancer (*n* = 27), transcriptomic data were downloaded from GEO database under GSE135222 and only the clinical information of PFS was available [22]. In addition, in nivolumab treatment cohort, pre/on therapy biopsies of 68 patients were analyzed by transcriptome sequencing, which was available on GEO database (GSE91061). We selected only the pretreatment samples with clinical information for further analysis [23]. Tumor response for all patients was defined by RECIST v1.1.

The corresponding clinical data from these patients were retrieved and manually arranged through the following two main methods: (1) utilizing the GEOquery R package or directly downloading on the GEO website through the corresponding accession number and (2) from the supplementary materials of the corresponding literature. The related authors were contacted for further information when necessary. Details of ethical approval and informed consent for all studies can be found in corresponding publications.

### Identification of differentially expressed genes and functional annotation

The DEGs between GC and normal gastric tissues were screened via the edgeR package [37]. The significant criteria for determining DEGs were set as the false discovery rate < 0.05 and the |fold change| > 2. Gene annotation enrichment analysis for GO and reactome pathway using the clusterProfiler R package [38] was conducted on senescence genes. GSEA was performed through clusterProfiler R package. And the genes were ordered according to the fold change of gene expression between the GC and normal tissue.

### Genomic data analysis

The somatic mutation data were acquired from TCGAmutations R package [39]. Besides, CNV and somatic mutation data in the meta-cohort were acquired from the cBio Cancer Genomics Portal (cBioPortal, http://www.cbioportal.org/) [40, 41]. The mutation landscape was depicted using the functions of maftools R package [42]. Genomic features of TCGA datasets, including clonal detection score, aneuploidy score, mutation density, etc., were obtained from the published research [3]. And data about the molecular subtypes of TCGA cohorts were also retrieved where available [43] (Table S3).

### Single-cell RNA-seq data analysis

In order to further portray senescence microenvironment in GC at single-cell level, we utilized the single-cell RNA (scRNA) data of gastric tissues previously published including nonatrophic gastritis, chronic atrophic gastritis, intestinal metaplasia, and GC [44]. The analytical pipeline of scRNA data as previously described [45]. Briefly, the raw count matrices of scRNA data were processed by the Seurat R package in R software [46]. Then, followed by quality control to filter low-quality cells, cells expressed lower than 400 genes or higher than 7000 detected genes, and more than 20% of all gene counts mapped to mitochondrial or ribosomal genes were removed as mentioned by the corresponding authors [44]. Then, the single-cell gene-expression data were normalized by using “NormalizeData” function with setting normalization method “LogNormalize” in the R package Seurat. We then utilized “ScaleData” function to regress out cell–cell variation driven by batch, the gene counts, as well as mitochondrial and ribosomal gene expression.

Furthermore, the highly variable genes were selected for the dimension reduction by “FindVariableFeatures” function in the Seurat package. Then, we used the “RunPCA” function in Seurat to perform the PCA. To establish the numbers of principle components representing the variance of cells, the “ElbowPlot” function was implemented, and 20 significant principal components were identified for further analysis.

We then used the “FindNeighbors” function in Seurat to assemble cells based on a graph-based clustering approach. Then, the “FindCluster” function with the resolution parameter 2 was implemented to identify the number of cell clusters. We utilized the “FindAllMarkers” function to find differentially expressed genes by comparing each cluster of cells with all other cells, and annotated cell clusters based on the expression of curated known cell markers.

### Unsupervised clustering for the senescence subtypes

To characterize senescence signature, Unsupervised clustering analysis was applied to identify distinct clusters based on the expression of CSGs. We adopted the function of the pheatmap R package based on the Euclidean distance and Ward’s linkage method to classify patients unsupervisedly. The number of clusters and their stability were determined by the NbClust package, which provided 30 indices for determining the number of clusters and proposed the best clustering scheme [47].

### Characterization of TME

To quantify the relative abundance of each cell infiltration in the GC, we used the single-sample GSEA (ssGSEA) algorithm. The gene sets for analyzing each immune cell type were obtained from the study of Charoentong et al. [48]. The enrichment scores calculated by ssGSEA analysis were utilized to represent the relative abundance of each type of immune cell [49, 50].

To investigate the difference of the biological process in different samples, GSVA analysis was performed using GSVA R packages. GSVA is a gene set enrichment method to estimate the variation in the pathway and biological process activity among different samples in a nonparametric and unsupervised manner [51]. The gene sets about senescence and other biological processes were downloaded from MSigDB of the Broad Institute for running GSVA analysis (Table S1) [52]. In addition, the gene sets that contained genes associated with TME, including the angiogenesis signature [53], antigen processing machinery (APM), DNA damage repair response, EMT, pan-fibroblast TGFβ response signature, and TGFβ pathway, were retrieved from a previous research [21].

### Development and validation of the senescence scoring and the prognostic nomogram

To dissect the senescence signature of individual tumor for better clinical utility, we developed a senescence score termed as senescore through LASSO machine learning algorithms. The specific process was as follows. The CSGs were put into the Cox regression model with LASSO penalty for analysis using the glmnet R package [54]. Genes of the model were established by the appropriate value of the penalty parameter *λ*, which were determined by tenfold cross-validations in TCGA-STAD cohort. The senescence risk score model was produced by integrating the expression level of selected genes and their corresponding coefficients derived from the prognostic model analyses [55], as follows: senescence risk score = (0.0394 × ADH1B) + (0.0515 × IL1A) + (−0.1386 × TNFAIP2) + (−0.0473 × EZH2) + (0.0100 × SPARC) + (0.1513 × SERPINE1). In order to make a positive consistency of senescence score and senescence features, we reformulated the senescence risk score as the senescore [56]:

$$\mathrm{Senescore} = e^{ - \mathop {\sum}\limits_{i = 1}^n {\beta _i \ast x_i} }$$

*β*_*i*_ represents the regression coefficient of each gene in Lasso regression model and *X*_*i*_ shows the gene-expression value of each gene.

The prognostic nomogram was generated based on the senescore using the rms R package and externally validated in the GSE66229 cohort. We conducted 3- and 5-year OS calibrations to determine the predictive accuracy of the nomogram model. The concordance index (C-index) and ROC curve were also used to evaluate the predictive accuracy of the model, and the time-dependent ROC curve was made using survivalROC R package.

### Multiplex immunofluorescence immunohistochemistry and imaging

A TMA spotted with tumor samples from 90 GC patients (HStmAde180Sur05) was purchased from Shanghai Outdo Biotech Co. Ltd. A total of 190 cores on the slide consisted of 90 cases of gastric cancer tissue and paired normal gastric tissue. The detailed clinical information was summarized in Table S7. All tissues were collected in accordance with the ethical standards with the donor being informed completely and with their consent, from National Human Genetic Resources Sharing Service Platform: 2005DKA21300.

For multiplex immunofluorescence staining [57], the Opal 7-color manual IHC kit (PerkinElmer, cat. No. NEL811001KT) was used according to the manufacturer’s instructions, and the molecules panel, which consisted of six antibodies including anti-IL1A (Proteintech, cat.No.16765-1-AP, 1:250 dilution), anti-ADH1B (Proteintech, cat.No.17165-1-AP, 1:500 dilution), anti-SERPINE1 (ABclonal, cat. No. A14758, 1:200 dilution), anti-SPARC (ABclonal, cat. No. A14494, 1:200 dilution), anti- EZH2 (ABclonal, cat. No. A19577, 1:200 dilution), and anti-TNFAIP2 (Signal Antibody, cat.No.40163, 1:200 dilution), was conducted on the same slide. In brief, the slides were baked at 65 °C for 1 h. Deparaffinization with xylene for 15 min three times was followed by rehydration in an ethanol series. Antigen retrieval was performed in the EDTA antigen retrieval buffer (pH 8.0) using microwave treatment, and the nonspecific binding was blocked with the blocking buffer for 10 min at room temperature. After that, the slide was stained with antigen-specific primary antibodies for 1 h at room temperature or overnight at 4 °C, following by Opal Polymer Horseradish Peroxidase for 10 min at room temperature. Then, a specific fluorochrome from the Opal 7-color manual IHC kit for each primary antibody was used to visualize the respective antigens. For each consecutive antibody staining, the antigen retrieval step, based on microwave treatment like the prior operation, was performed, which allowed for the removal of prior primary and secondary antibodies while the fluorophore remained covalently bonded to the tissue antigen.

The stained slides were scanned using the Vectra Polaris Automated Quantitative Pathology Imaging System (PerkinElmer) by a 20× objective lens. Inform automated image analysis software (PerkinElmer) was used for batch analysis of multispectral images based on the specific algorithms as previously described [58]. Multispectral images were decomposed into their single component by spectral unmixing using a digital spectral library consisting of spectral profiles of each of the fluorophores.

Due to the stripping the slides and limitation of scanning technique, 80 tissue samples were involved in final analysis. The IHC images of each molecular staining results were scored twice by the clinician. The protein scoring system was based on the intensity and extent of the staining as previously described [59]. The staining intensity was classified as 0 (negative), 1 (weak), 2 (moderate), or 3 (strong). The staining extent was dependent on the percentage of positive area: 0 (<5%), 1 (5–25%), 2 (26–50%), 3 (51–75%), or 4 (>75%). Each protein score was based on the stain intensity and extent scores, and then normalized with a *z*-score. Finally, the senescore was calculated on the basis of the protein expression scores of IL1A, ADH1B, TNFAIP2, SERPINE1, SPARC, and EZH2 (Table S7).

### Statistical analysis

The distribution of the variables was tested by the Shapiro–Wilk test. For the comparison of two groups, statistical significance of variables was analyzed by the unpaired Student’s *t* test or the Wilcoxon rank-sum test. The Kruskal–Wallis test was applied to evaluate significant differences when comparing more than two groups. Correlations coefficients between CSGs were computed by Spearman at the transcriptomic level.

The prognosis genes were determined by univariate Cox regression analysis. The survival curves for the prognostic analysis were generated via the Kaplan–Meier method and the log-rank test was used to compare the statistical differences in survival between two groups. To analyze the significance of senescore to predict clinical prognosis, the cut-off values of senescore were establish by the median of the data in each separate dataset. Alternatively, to attenuate the effect of the group method on prognosis, the cut-off point was also determined using “surv-cutpoint” function in the survminer R package to divide patients into high- and low-senescore groups in the immunotherapy and chemotherapy cohort. The independent prognostic factor was ascertained through a multivariable Cox regression model. The forest plot was employed to visualize the results of multivariate prognostic analysis using the survminer R package. All heatmaps were generated by the function of the pheatmap R package. The *p* values were two-sided, with less than 0.05 as statistically significance. All statistical analyses were conducted in R language software (version 3.6.1).

## Supplementary information

Supplementary Figure Legends

Supplemental figures

Table S1

Table S2

Table S3

Table S4

Table S5

Table S6

Table S7

## Data Availability

The data that support findings of this study are available in the GEO (https://www.ncbi.nlm.nih.gov/geo/) under accession numbers GSE26253, GSE26899, GSE26901, GSE28541, GSE13861, GSE66229, GSE15459, GSE34942, GSE84437, GSE57303, and GSE29272, as well as TCGA (https://portal.gdc.cancer.gov) database.
