# Supplementary material for: Senescence as a dictator of patient outcomes and therapeutic efficacies in human gastric cancer
Source: Cell Death Discov. 2022 Jan 10;8:13. doi: 10.1038/s41420-021-00769-6 (PMC8748965; doi:10.1038/s41420-021-00769-6)
Supplement: Supplementary file 1 — Supplementary Figure Legends [file 41420_2021_769_MOESM1_ESM.docx]

**Supplementary Figure Legends**

**Figure S1. Identification of the senescence character in GC. A.** GSEA plot showed that GO term associated with senescence was significantly enriched in GC compared with normal tissues in TCGA-STAD cohort. **B.** GSEA plot exhibited the enriched pathway associated with senescence based on GC versus normal gastric tissues in GSE29272 cohort. **C-D.** Heatmap (**C**) and volcano plot (**D**) demonstrating differentially expressed genes between GC and normal tissues in TCGA-STAD cohort. **E.** Bar plot showing enriched KEGG pathways of differentially expressed genes.

**Figure S2. Transcriptomic and genomic features of CSGs. A.** UMAP plot showed single-cell transcriptomic profiling of different sample distribution of gastric normal tissues and GC. **B.** Senescence signature enrichment displayed in different cells from gastric tissues of non-atrophic gastritis and chronic atrophic gastritis. **C.** Mutation landscape of CSGs in the meta-cohort. **D.** The mutation co-occurrence and exclusion analyses for CSGs in TCGA-STAD dataset. Co-occurrence, violet; exclusion, wheat. **E.** The relative expression of 17 CSGs between GCs and paired normal tissues in GSE66229 dataset. Box and whisker plots show all data points, and the box represents the second and third quartiles and median. Significance was analyzed using the Wilcoxon rank-sum test. The asterisks represented the statistical p value (*P < 0.05; **P < 0.01; ***P < 0.001).

**Figure S3. Validation of the senescence subtypes.** **A.** NbClust test of unsupervised clustering. **B.** The relative expression of CSGs between two clusters. The asterisks represented the statistical p value (the unpaired Student t test, *P < 0.05; **P < 0.01; ***P < 0.001). **C.** Unsupervised clustering of patients in validation cohort1 to validate senescence subtypes. GEO accession numbers of cohorts were used as sample annotations. **D.** Kaplan-Meier curves indicated that senescence subtypes were markedly related to OS in 1484 patients of validation cohort1.P value was calculated by Log-rank test.

**Figure S4. Clinical characteristics of senescence subtypes. A-E.** The stacked barplots showing the fraction of the TNM stage (**A**), age distribution (**B**), histological classification (**C**), molecular subtypes (**D**), and MSI status (**E**) in two clusters. Statistical significance was tested by two-sided Fisher’s exact test. **F.** Box plot showing the significant difference of the clonal detection score between two senescence subtypes. P value was calculated by Wilcoxon test.

**Figure S5. Development and characterization of the senescore in TCGA-STAD cohort. A.** Partial likelihood deviance of different numbers of variables revealed by the LASSO regression model. Tuning parameter (λ) selection used ten-fold cross-validation via the maximum criteria. The red dots represent the partial likelihood deviance values, the grey lines represent the standard error (SE), the two vertical dotted lines on the left and right, respectively, represent optimal values by minimum criteria and 1-SE criteria. **B.** LASSO coefficient profiles of the 17 senescence core genes. **C.** The relation between the overall survival status and distribution of the senescore. **C.** Time‐dependent ROC analysis was used to evaluate the accuracy of the prognostic nomogram. **D-E.** Calibration plots comparing predicted and actual survival probabilities at 3‐ and 5‐year OS. The blue dashed lines represent the ideal predictive model, and the red solid line represents the observed model. Red dots represent nomogram-predicted probabilities; blue crosses represent the bootstrap-corrected estimates; and error bars represent the 95% CIs of these estimates. **F-G.** The distinctions of the senescore in different MSI status (**F**) and histological classification (**G**) in TCGA-STAD cohort. The statistical significance between two group was analyzed by the Wilcoxon rank-sum test. The Kruskal-Wallis test was applied to evaluate significant differences among all the groups.

**Figure S6. Validation of the senescore for clinical outcome in different cohorts. A.** The differences of the senescore among histological classification in ACRG cohort. The statistical significance between two group was analyzed by the Wilcoxon rank-sum test. The Kruskal-Wallis test was applied to evaluate significant differences among all the groups. **B-G.** Survival analysis of senescore in all gastric cancer patients in our study (B), TCGA-COADREAD (B)**,** colon cancer GSE39582 (C), TCGA-ESCA (E), TCGA-LIHC (F), and TCGA-PAAD(G). The log-rank P value was indicated.

**Figure S7. Validation of the senescore for therapeutic efficacies in different cohorts A.** Disease-free survival analyses for patients in ACRG cohort stratified by both the senescore and treatment with adjuvant chemotherapy using Kaplan-Meier curves. **B.** Kaplan–Meier plots for OS of patients in adjuvant chemotherapy cohort who received adjuvant chemotherapy and those who did not in each senescore group. P values were obtained using the log-rank test. **C** Kaplan-Meier curves showed survival differences of OS between low and high senescore patients in an anti-PD1/PD-L1 therapy cohort. **D.** Kaplan-Meier plot of the overall survival of immunotherapy patients stratified by the senescore quartile. **E.** Boxplot showed the difference of senescore in distinct therapy response group. P value was calculated by the Wilcoxon rank-sum test. **F.** Scatterplot showing the correlations of the senescore with tumor mutation burden. R coefficient and p value was calculated by Pearson correlation test. ADCT: adjuvant chemotherapy.

**Figure S8. Multiplex immunofluorescence immunohistochemistry profiling for prognostic analysis of the senescence biomarkers in tissue microarray of GC. A-F.** Survival analysis for protein expression quantification of ADH1B (A), SERPINE1 (B), SPARC (C), TNFAIP2 (D), IL1A(E) and EZH2 (F) in tissue microarray of GC. P value was calculated by Log-rank test.

**Table S1. Information of senescence gene sets**

**Table S2. Summary of patient's information of cohorts for clinical outcomes**

**Table S3. Detailed information of TCGA-STAD cohort in this study.**

**Table S4. Details about the datasets used in this study**

**Table S5. Detailed information of chemotherapy cohorts in this study**

**Table S6. Detailed information of immunotherapy cohorts in this study**

**Table S7. Detailed information of tissue microarray cohort in this study**
