# Supplementary figures and images for: Senescence as a dictator of patient outcomes and therapeutic efficacies in human gastric cancer
Source: Cell Death Discov. 2022 Jan 10;8:13. doi: 10.1038/s41420-021-00769-6 (PMC8748965; doi:10.1038/s41420-021-00769-6)

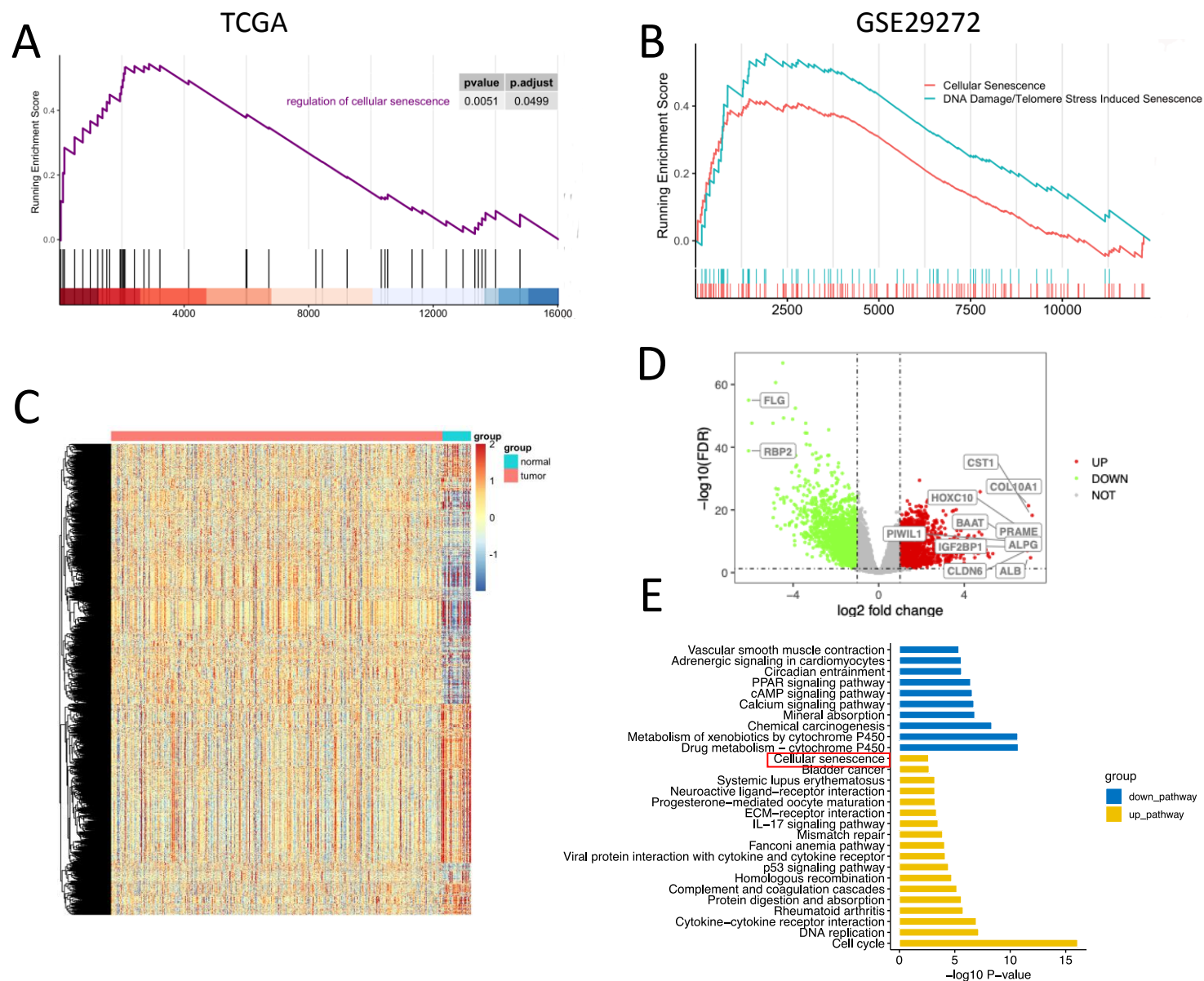

Figure S1

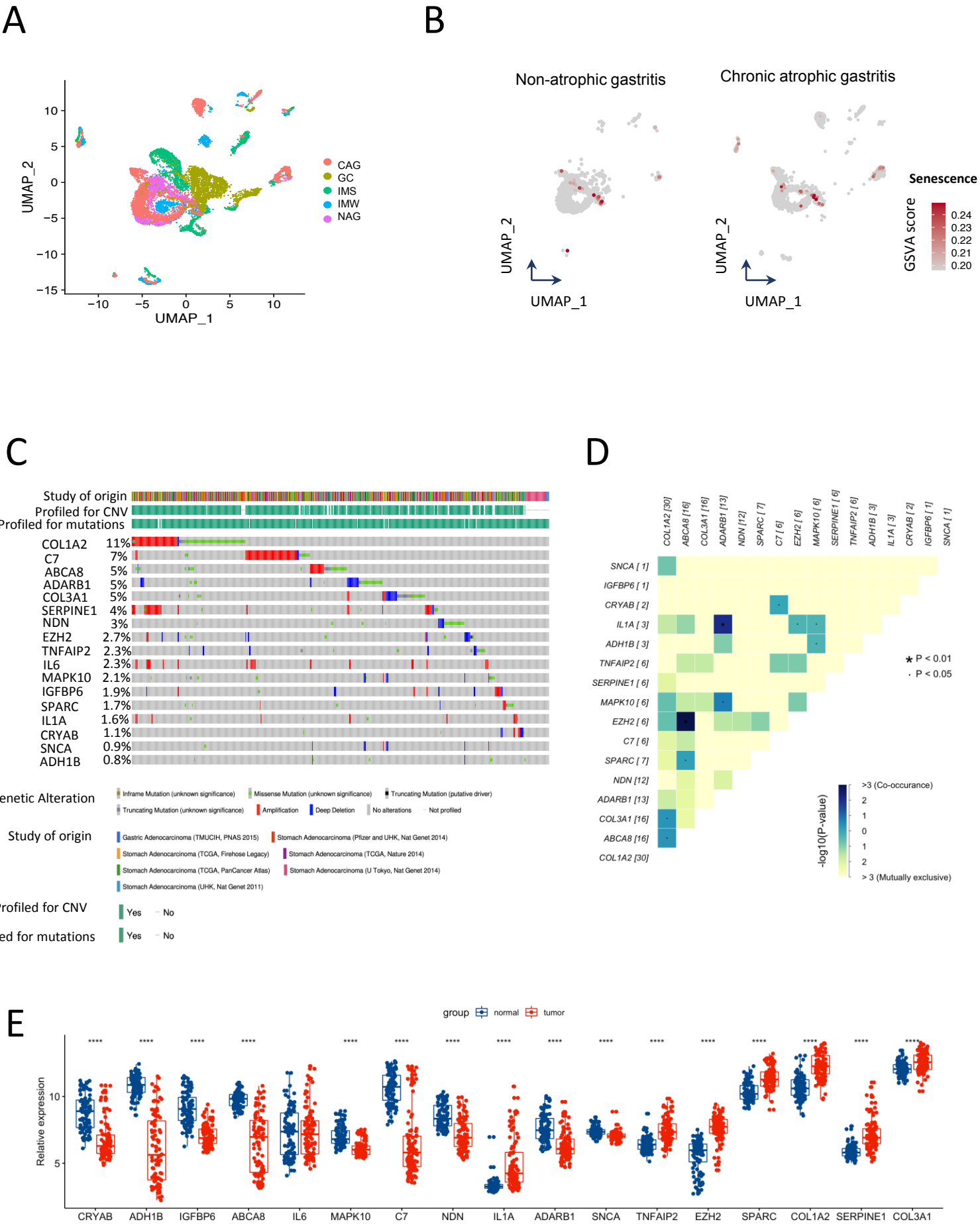

Figure S2

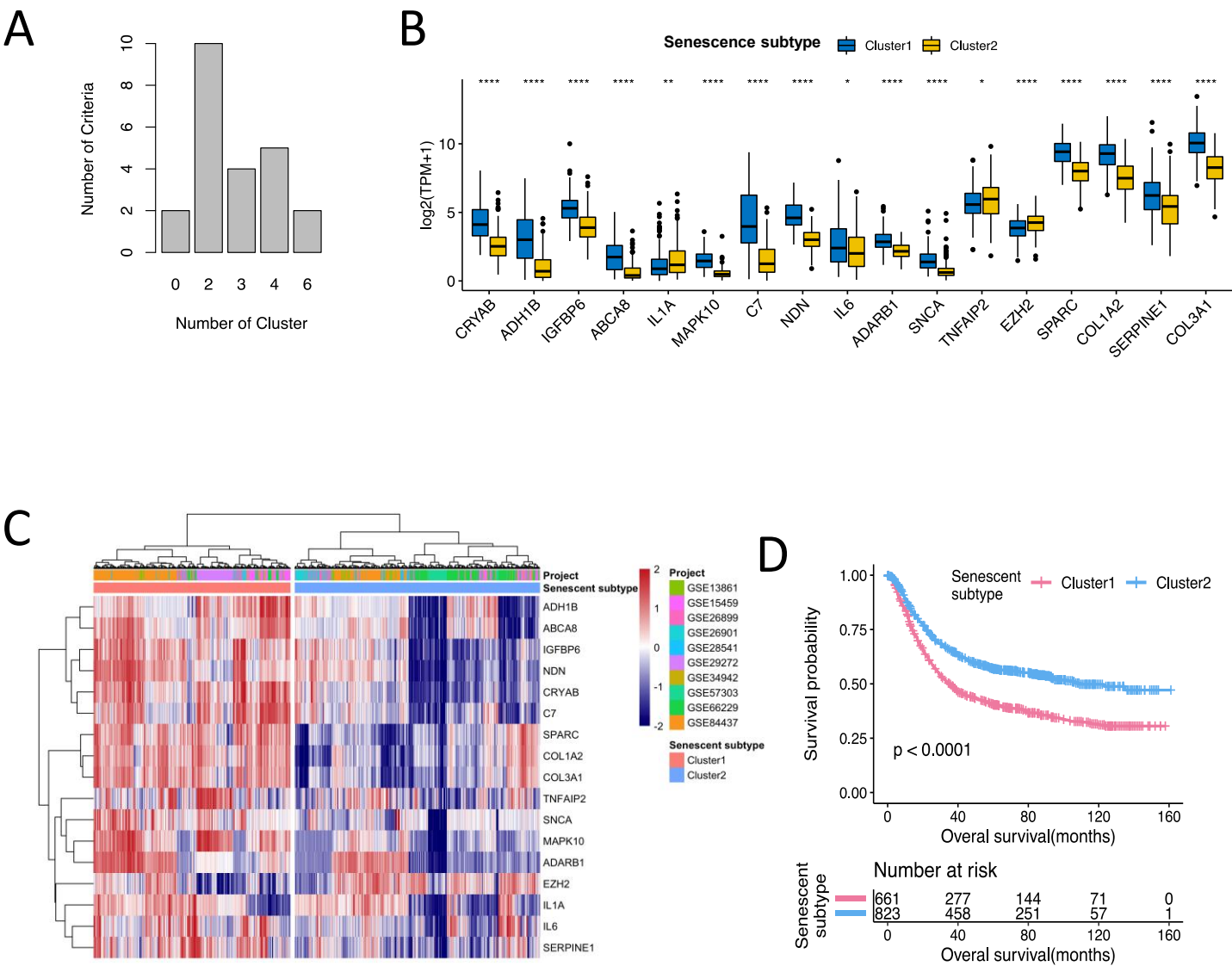

Figure S3

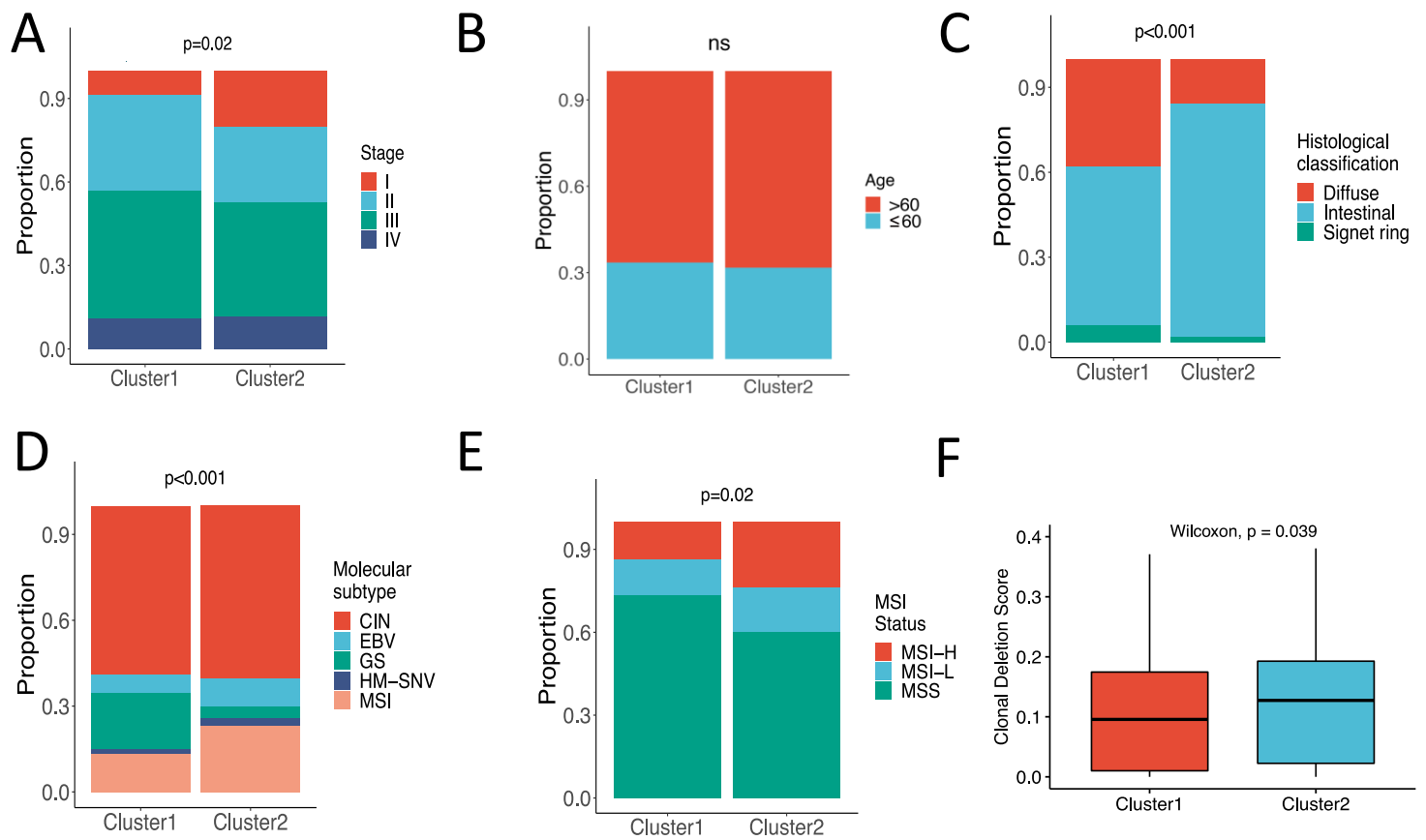

Figure S4

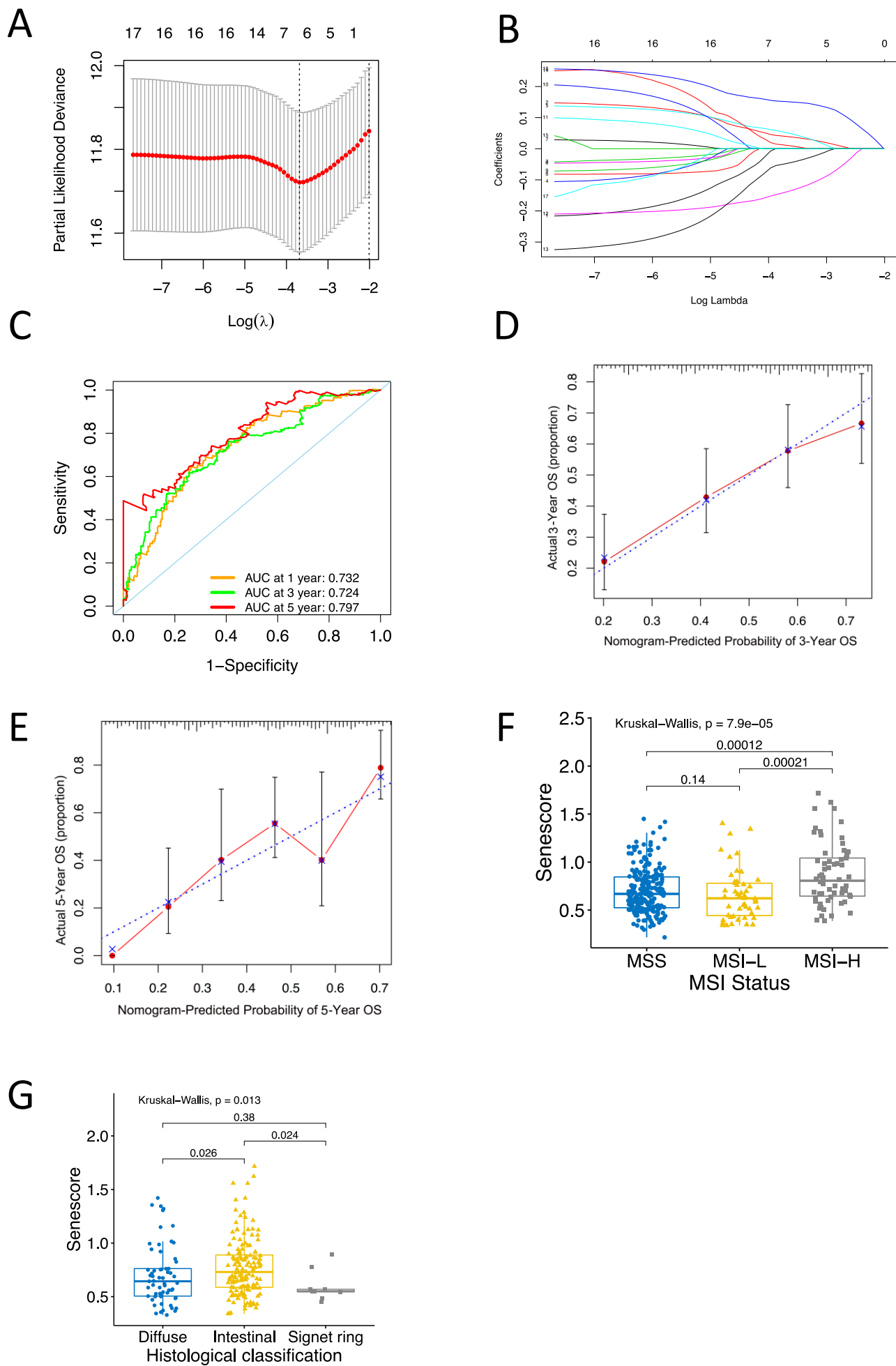

Figure S5

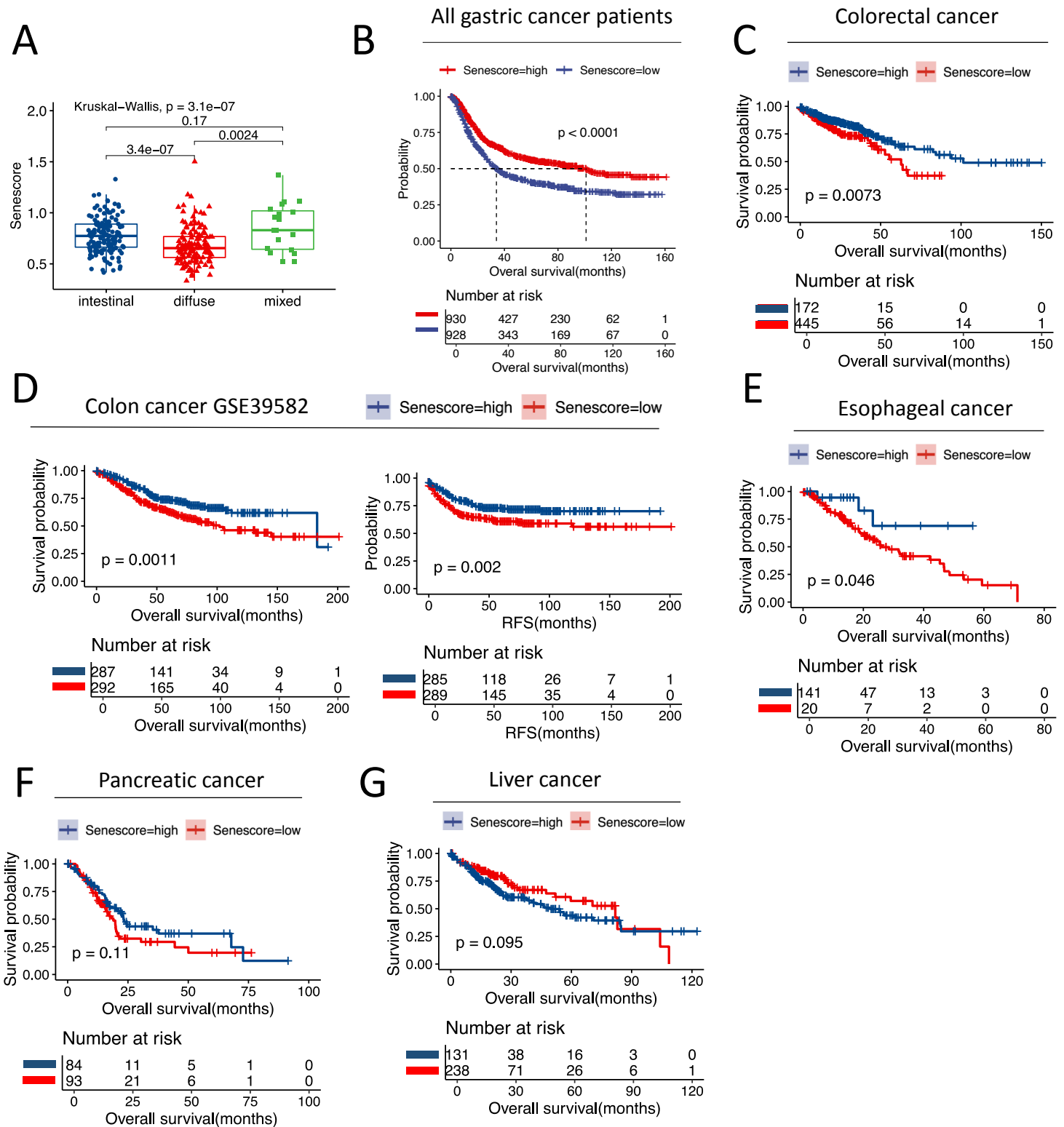

Figure S6

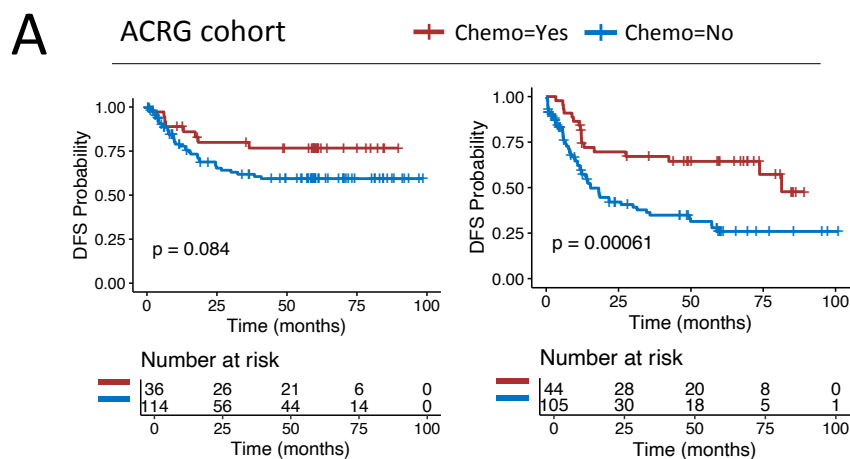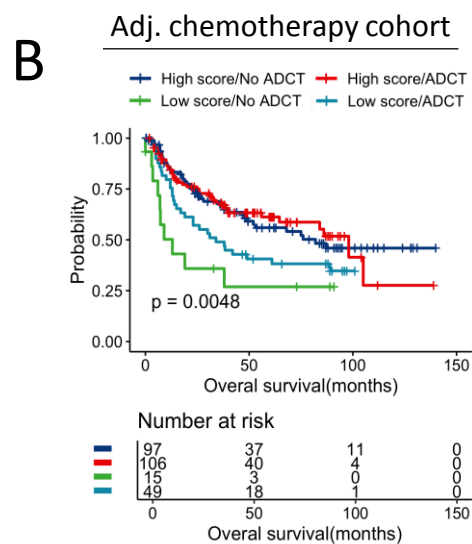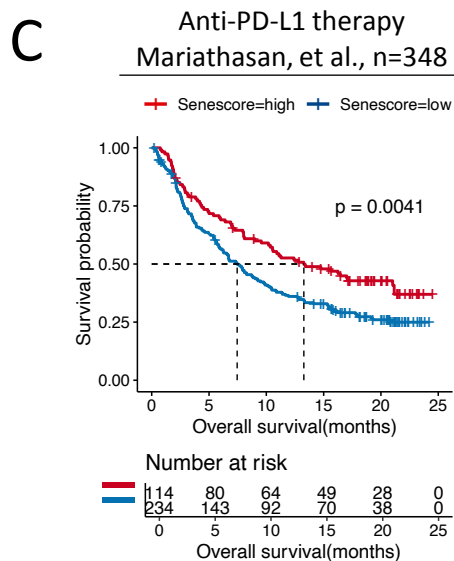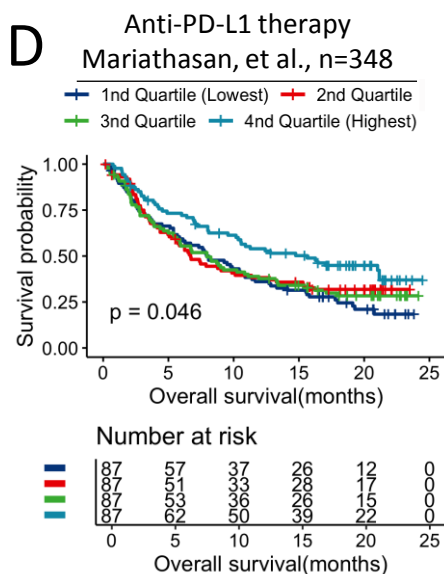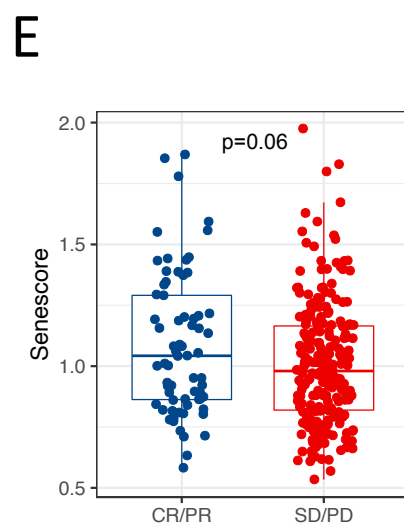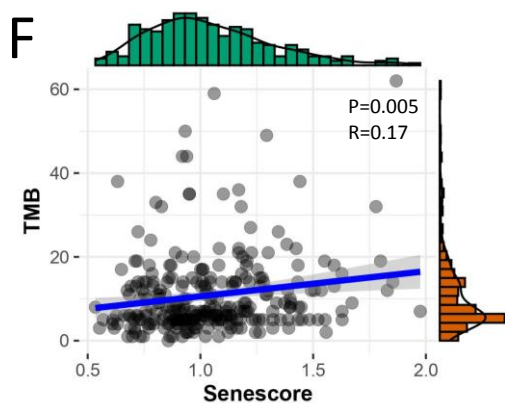

Figure S7

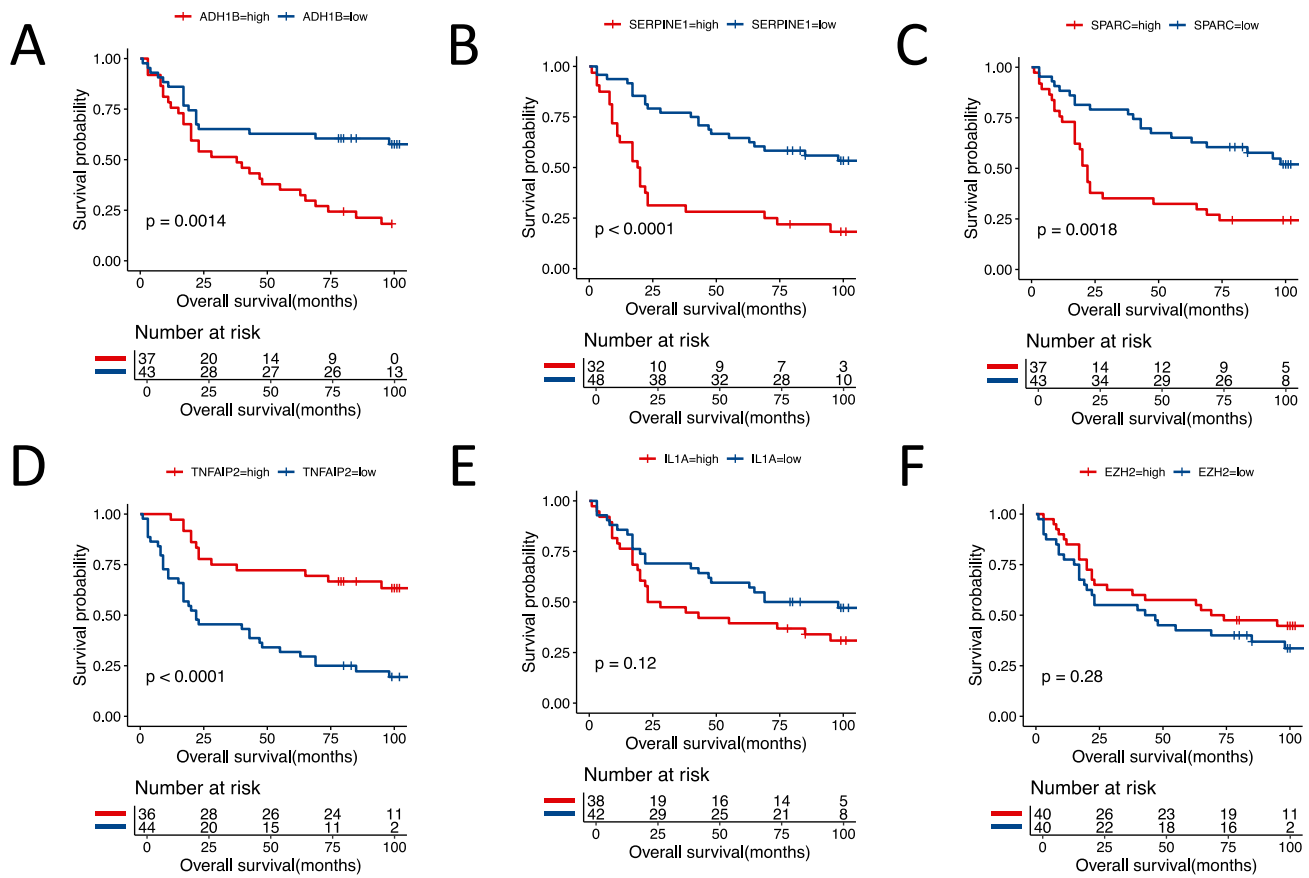

Figure S8

Supplement: Supplementary file 2 — Supplemental figures [file 41420_2021_769_MOESM2_ESM.pdf]
